# Characteristics of Human Metapneumovirus Infection Compared to Respiratory Syncytial Virus and Influenza Infections in Adults Hospitalized for Influenza-Like Illness in France, 2012–2022

**DOI:** 10.1093/infdis/jiaf082

**Published:** 2025-07-16

**Authors:** Paul Loubet, Salomé Guitton, Simon Rolland, Louise H Lefrancois, Liem Binh Luong Nguyen, Philippe Vanhems, Fabrice Laine, Florence Galtier, Xavier Duval, Bruno Lina, Martine Valette, Giséle Lagathu, Vincent Foulongne, Nadira Houhou-Fidhou, Anne Sophie L’Honneur, Fabrice Carrat, Laurence Meyer, Christine Durier, Odile Launay

**Affiliations:** Virulence Bactérienne et Infections Chroniques, INSERM U1047, Univ Montpellier, Service des Maladies Infectieuses et Tropicales, CHU Nîmes, Nîmes; Inserm, F-CRIN, Innovative Clinical Research Network in Vaccinology (I-REIVAC), Paris; Inserm SC10-US19, Villejuif; Service des maladies infectieuses et tropicales, Centre Hospitalier Universitaire de Brest; Inserm, F-CRIN, Innovative Clinical Research Network in Vaccinology (I-REIVAC), Paris; Université Paris Cité, Inserm, Centre d’Investigation Clinique Cochin Pasteur, Assistance Publique Hôpitaux de Paris, Hôpital Cochin, Paris; Service d’Hygiène, Epidémiologie et Prévention, Hospices Civils de Lyon; Centre d’Investigations Cliniques, Inserm, Unité mixte de recherche, Centre d’Investigation Clinique 1414, Hôpital Pontchaillou, Rennes; Inserm, Centre d'Investigation Clinique 1411, Centre Hospitalier Universitaire Montpellier, Hôpital Saint Eloi, Montpellier; Université Paris Cité and Université Sorbonne Paris Nord, Inserm 1137, Infection, Antimicrobials, Modelling, Evolution, Paris; Inserm Centre d’Investigation Clinique 1425, Assistance Publique Hôpitaux de Paris, Hôpital Bichat, Paris; Laboratoire de Virologie, Hospices Civils de Lyon, Institut des Agents Infectieux, Centre National de Référence des virus Respiratoires France Sud, Hôpital de la Croix-Rousse, Lyon; Laboratoire de Virologie, Centre Hospitalier Universitaire de Rennes, Hôpital Pontchaillou, Rennes; Laboratoire de Virologie, Centre Hospitalier Universitaire de Rennes, Hôpital Pontchaillou, Rennes; Service de Virologie, Centre Hospitalier Universitaire Montpellier, Hoôpital Saint Eloi, Montpellier; Laboratoire de Virologie, Hoôpital Bichat Claude Bernard, Paris; Service de Virologie, Hoôpital Cochin, Paris; Sorbonne Université, Inserm, Institut Pierre Louis d’Epidémiologie et de Santé Publique, Assistance Publique Hôpitaux de Paris, Hôpital Saint Antoine, Paris; Inserm SC10-US19, Villejuif; Paris Saclay University, Inserm U1018, Assistance Publique Hôpitaux de Paris, Hôpital Bicêtre, France; Inserm SC10-US19, Villejuif; Inserm, F-CRIN, Innovative Clinical Research Network in Vaccinology (I-REIVAC), Paris; Université Paris Cité, Inserm, Centre d’Investigation Clinique Cochin Pasteur, Assistance Publique Hôpitaux de Paris, Hôpital Cochin, Paris

**Keywords:** human metapneumovirus, influenza, respiratory syncytial virus, adults, hospitalization

## Abstract

**Background:**

We aimed to compare the characteristics of human metapneumovirus (hMPV) infection with influenza A and B virus (FLUV) and respiratory syncytial virus (RSV) infections in adults hospitalized with influenza-like illness (ILI).

**Methods:**

We conducted a post hoc analysis of adult patients hospitalized with community-acquired ILI who were enrolled in the FLUVAC study at 5 French referral hospitals from 2012 to 2022.

**Results:**

At least 1 respiratory virus was detected in 3620 of 6618 patients (55%), including FLUV (1524/3620 [42%]), RSV (248/3620 [7%]), and hMPV (162/3620 [5%]). hMPV^+^ patients, when compared to FLUV^+^ patients were more likely to develop at least 1 complication (60% [86/143] vs 50% [716/1435]; *P* = .02), especially acute heart failure, which occurred twice as often in hMPV^+^ during the hospital stay (22% [32/143] vs 11% [160/1434]; *P* < .001). The rates of respiratory (30% [43/143] vs 32% [70/216]; *P* = .73) or cardiac (22% [32/143] vs 15% [33/216]; *P* = .09) complications did not differ between hMPV^+^ and RSV^+^ patients. The in-hospital all-cause death rate was similar among all 3 populations (4% hMPV^+^, 4% FLUV^+^, and 5% RSV^+^).

**Conclusions:**

Hospitalized hMPV infections affect older patients with multiple chronic conditions who face frequent cardiac and pulmonary complications during hospitalization more frequently than with influenza and similar to RSV.

Human metapneumovirus (hMPV) is a significant concern for individuals of all ages, particularly those with chronic conditions [1]. It can manifest with various clinical presentations, from mild upper respiratory tract symptoms to severe pneumonia [2, 3]. However, there is a notable scarcity of data on its frequency and outcomes in older patients. This is mainly due to the underestimation of its incidence, which results from the lack of testing in ambulatory care and the variability in testing practices in hospital settings.

As specific antiviral therapies and vaccines targeting hMPV are under development [4–6], better characterization of patients’ profiles and outcomes is warranted.

The objectives of the study were to (1) provide a comprehensive description of the characteristics and outcomes of hMPV infection and (2) compare the characteristics and outcomes of hMPV, influenza A and B (FLUV), and respiratory syncytial virus (RSV) infections in a large number of adult patients hospitalized for community-acquired influenza-like illness (ILI) in France from 2012 to 2022.

## METHODS

### Study Design

We performed a post hoc analysis of the FLUVAC study. FLUVAC is a prospective case-control and test-negative design study evaluating influenza vaccine effectiveness on influenza-associated hospitalization conducted in 5 referral hospitals in France [7]. All adults hospitalized for at least 24 hours for community-acquired ILI during the influenza circulation period during the years 2012–2013 to 2019–2020 and, due to the coronavirus disease 2019 (COVID-19) pandemic, year-round in 2020–2021 and 2021–2022, with symptom onset <7 days before screening, were included in FLUVAC. ILI was defined according to the European Centre for Disease Prevention and Control definition [8] as a combination of the following: (1) at least 1 of the following systemic symptoms: fever or feverishness, headache, myalgia, or malaise; and (2) at least 1 of the following respiratory symptoms: cough, sore throat, or dyspnea. Patients with contraindications to influenza immunization, those who had previously tested positive for FLUV in the same season, and those without French social security coverage were excluded. Each participant was interviewed, and nasopharyngeal samples were obtained at enrollment to screen for FLUV and other respiratory viruses.

We included in this analysis all the patients for whom virologic results on respiratory viruses were available from 10 influenza seasons (2012–2013 to 2021–2022). Patients with coinfections were excluded. A sensitivity analysis was performed without the exclusion of coinfections.

### Outcomes

We first defined the prevalence of confirmed hMPV, FLUV, and RSV infections in patients hospitalized with ILI. Next, we compared the patients’ characteristics, the clinical presentation of the ILI episode, the complications (not present at admission), and outcomes among laboratory-confirmed hMPV, RSV, and FLUV infections.

### Microbiological Data

All nasopharyngeal swabs were tested for respiratory viruses (adenovirus, human bocavirus, human coronaviruses [229E, NL63, OC43, HKU1], hMPV, parainfluenza viruses 1–4, picornavirus, and RSV) using multiplex reverse-transcription polymerase chain reaction (Allplex RP1, RP2, and RP3 kits, Seegene, Seoul, South Korea). In 2020–2021 and 2021–2022, severe acute respiratory syndrome coronavirus 2 (SARS-CoV-2) detection was systematically added in local laboratories using different kits. Clinical bronchoalveolar lavage fluid samples and tracheal aspirates were also tested if available. Other pathogen testing, including bacteria, was left to the physicians’ discretion.

### Statistical Analysis

Continuous variables were expressed as mean and standard deviation or median and interquartile range (IQR), and qualitative variables as numbers and percentages. Qualitative variables were compared using χ² and Fisher exact tests, as appropriate. The Wilcoxon rank-sum test compared continuous variables. Missing data for each variable were excluded from the denominator.

For hMPV infection (positive vs negative), the analyzed population included individuals tested for hMPV, regardless of whether they were tested for other viruses. However, the results indicate either hMPV^+^ (excluding coinfections) or hMPV^–^, irrespective of the status of any other tested virus. Univariable and multivariable analyses were performed using a conditional logistic regression model with stratification by season. The analyses included factors such as sex, age, smoking status, presence of cardiorespiratory or other comorbidities, immunosuppressive treatment, influenza vaccination in the same season, and a history of hospitalizations in the previous 12 months. Using conditional logistic regression models with season as the stratification variable allowed for estimating models conditional on each season and helped eliminate bias that may arise from higher hMPV rates in certain seasons. Results from regression models are expressed as crude odds ratios and adjusted odds ratios with 95% confidence interval (CI). A *P* value of <.05 was considered statistically significant. Univariate tests were used to compare hMPV^+^ and FLUV^+^ patients, hMPV^+^ and RSV^+^ patients, and hMPV^+^ and patients without any respiratory virus found. hMPV^+^ versus FLUV^+^ was also compared according to influenza vaccine status. Analyses were performed using R software (version 4.3.2).

### Ethics

The FLUVAC study (ClinicalTrials.gov identifier NCT02027233) followed Good Epidemiological and Clinical Practices in Clinical Research and the Declaration of Helsinki and was approved by the National Ethics Committee (CPP 2013/44). All study participants gave their informed consent for respiratory virus testing.

## RESULTS

**Figure 1.**
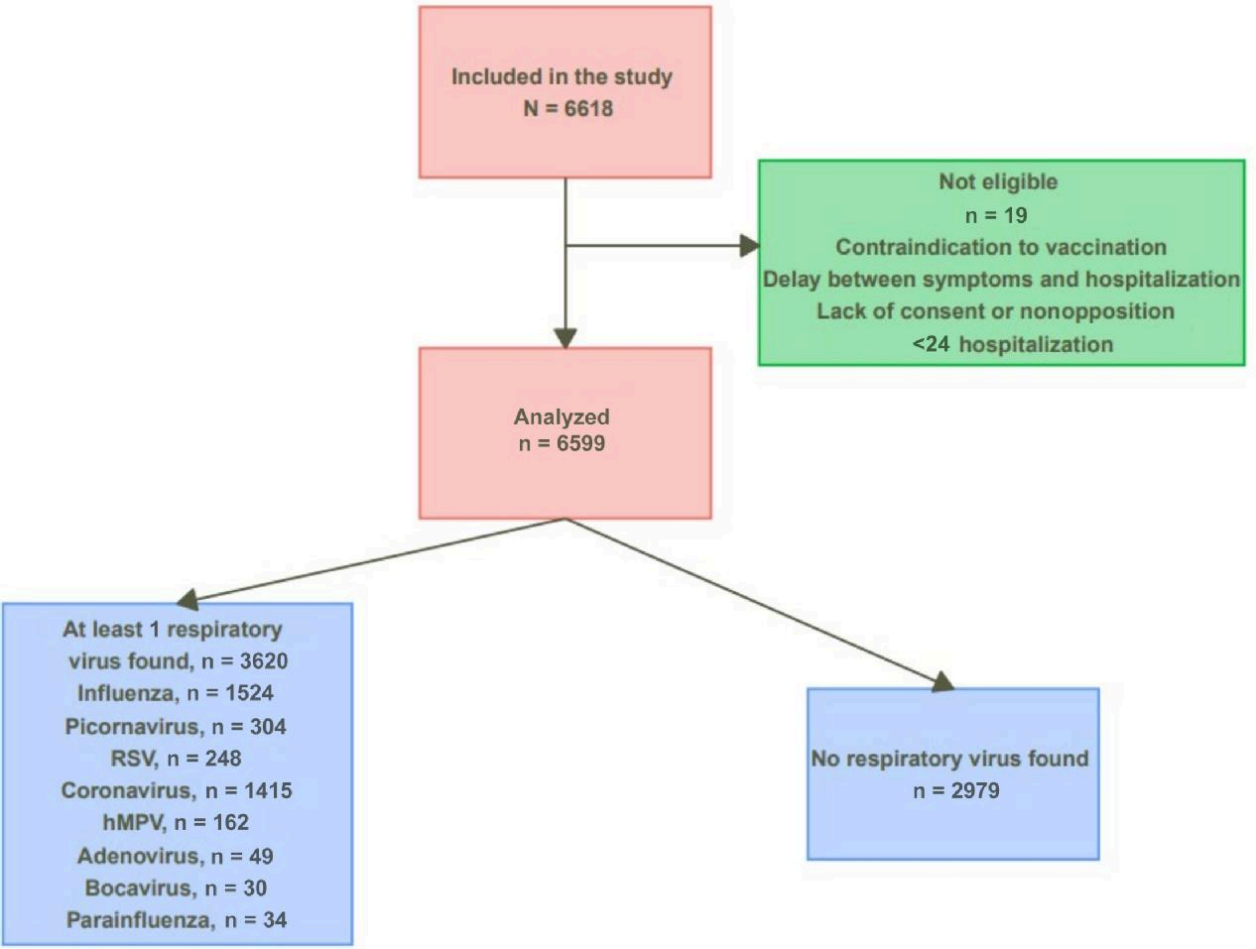
Study flowchart. Abbreviations: hMPV, human metapneumovirus; RSV, respiratory syncytial virus.

Of the 6618 patients included, 6599 were analyzed, of whom 3620 (55%) tested positive for at least 1 respiratory virus. Among positive patients, FLUV (1524 [42%, including 77% influenza A and 23% influenza B]), SARS-CoV-2 (1226 [34%]), picornavirus (304 [9%]), RSV (248 [7%]), and seasonal coronaviruses (189 [5%]) were the most prevalent viruses (Figure 1 and Table 1).

**Table 1.** Characteristics and Outcome of Hospitalized Patients Infected With Human Metapneumovirus, Influenza Virus, Respiratory Syncytial Virus, or No Respiratory Virus, 2012–2022, After Exclusion of Coinfections

Characteristic	hMPV^+^ (n = 143)	FLUV^+^ (n = 1435)	*P* Value	RSV^+^ (n = 216)	*P* Value	No Respiratory Virus Found (n = 2979)	*P* Value
Baseline characteristics							
Sex, female	82/143 (57)	712/1435 (50)	.08	114/216 (53)	.45	1332/2979 (45)	.003
Age, y, median (IQR)	78 (67–85)	71 (56–82)	<.001	74 (64–85)	.24	73 (59–84)	.003
Age group, y			<.001		.27		.004
18–49	9/143 (6)	268/1435 (19)		11/216 (5)		451/2979 (15)	
50–64	23/143 (16)	287/1435 (20)		49/216 (23)		538/2979 (18)	
65–74	28/143 (20)	283/1435 (20)		50/216 (23)		627/2979 (21)	
≥75	83/143 (58)	597/1435 (42)		106/216 (49)		1363/2979 (46)	
BMI, kg/m^2^, median (IQR)	24.7 (21.3–29.3)	24.9 (21.8–28.7)	.59	24.6 (21.6–28.9)	.52	25 (21–29)	.47
Smoking status			.19		.12		.21
Smoker	20/140 (14)	289/1397 (21)		44/212 (21)		566/2901 (20)	
Ex-smoker	41/140 (29)	377/1397 (27)		71/212 (33)		889/2901 (31)	
Nonsmoker	79/140 (56)	731/1397 (52)		97/212 (46)		1446/2901 (50)	
Chronic disease (at least 1)	116/143 (81)	1093/1433 (76)	.21	187/215 (87)	.14	2386/2955 (81)	1.0
Chronic respiratory disease	56/143 (39)	560/1435 (39)	1.00	113/216 (52)	.02	1315/2961 (44)	.23
Chronic heart disease	72/143 (50)	572/1435 (40)	.02	99/216 (46)	.45	1244/2960 (42)	.06
Diabetes	31/143 (22)	330/1435 (23)	.84	47/216 (22)	1.00	716/2961 (24)	.55
Chronic renal failure	19/143 (13)	185/1435 (13)	.90	36/216 (17)	.46	498/2961 (17)	.30
Cancer	17/143 (12)	209/1435 (15)	.45	44/215 (20)	.04	518/2960 (18)	.09
Cirrhosis	7/143 (5)	53/1433 (4)	.49	4/216 (2)	.12	144/2957 (5)	1.0
Immunosuppressive treatment	22/143 (15)	230/1430 (16)	.91	43/215 (20)	.33	450/2934 (15)	1.0
Influenza vaccination	81/142 (57)	615/1427 (43)	.004	126/213 (59)	.74	1562/2939 (53)	.60
Hospitalization in the previous 12 mo	59/143 (41)	589/1433 (41)	1.00	98/216 (45)	.45	1435/2932 (49)	.09
Presence of child <5 y in the household	7/143 (5)	115/1435 (8)	.25	15/216 (7)	.51	138/2943 (5)	.84
Clinical presentation							
Time from symptom onset to hospitalization, d, median (IQR)	2 (1–3)	2 (1–4)	.97	2 (1–3)	.85	2 (1–4)	.19
Symptoms							
Fever or feverishness	129/143 (90)	1280/1434 (89)	.89	183/215 (85)	.20	2421/2977 (81)	.01
Cough	125/143 (87)	1239/1434 (86)	.80	185/215 (86)	.75	1925/2977 (65)	<.001
Dyspnea	104/121 (86)	869/1184 (73)	.002	175/187 (94)	.03	2141/2526 (85)	.80
Weakness/malaise	14/143 (10)	352/1429 (25)	<.001	46/213 (22)	.004	651/2973 (22)	.001
Headache	28/143 (20)	405/1428 (28)	.02	47/214 (22)	.69	614/2969 (21)	.84
Myalgia	29/143 (20)	377/1423 (26)	.11	33/213 (15)	.26	597/2971 (20)	1.0
Sore throat	14/143 (10)	248/1427 (17)	.02	35/212 (17)	.09	334/2964 (11)	.71
Outcomes							
At least 1 complication during the hospital stay	86/143 (60)	716/1435 (50)	.02	133/216 (62)	.83	1521/2942 (52)	.05
Pneumonia	45/128 (35)	382/1365 (28)	.10	71/202 (35)	1.0	709/2376 (30)	.20
Respiratory failure	43/143 (30)	317/1435 (22)	.04	70/216 (32)	.73	556/2930 (19)	.002
Acute heart failure	32/143 (22)	160/1434 (11)	<.001	33/216 (15)	.09	344/2930 (12)	<.001
ARDS	9/143 (6)	124/1435 (9)	.43	24/216 (11)	.14	180/2930 (6)	.86
ICU admission	22/143 (15)	243/1435 (17)	.73	55/216 (25)	.03	534/2941 (18)	.44
Mechanical ventilation	11/89 (12)	105/760 (14)	.87	34/161 (21)	.09	276/2019 (14)	.87
Invasive	1/2 (50)	4/23 (17)	.37	3/10 (30)	1.0	21/145 (14)	.28
Noninvasive	1/2 (50)	21/23 (91)	.23	8/10 (80)	.46	131/146 (90)	.21
ECMO	0/89 (0)	3/760 (0)	1.0	0/161 (0)	1.0	5/2020 (0)	1.0
All-cause death	6/143 (4)	51/1433 (4)	.64	10/216 (5)	1.0	134/2956 (5)	1.0

Data are presented as No. (%) unless otherwise indicated. *P* values are for comparison between hMPV^+^ and FLUV^+^ or RSV^+^ or no respiratory virus found.

Abbreviations: ARDS, acute respiratory distress syndrome; BMI, body mass index; ECMO, extracorporeal membrane oxygenation; FLUV, influenza virus; hMPV, human metapneumovirus; ICU, intensive care unit; IQR, interquartile range; RSV, respiratory syncytial virus.

Overall, hMPV was found in 162 patients, which accounted for 2.5% (95% binomial CI, 2.1%–2.8%) of people with ILI symptoms and 4.5% (95% binomial CI, 3.8%–5.2%) of those who tested positive for at least 1 respiratory virus. Among the hMPV cases, 12% (19/162) were coinfection cases with at least 1 other virus (3 with influenza A virus, 1 with an untyped influenza virus, 7 with coronavirus [4 seasonal and 3 SARS-CoV-2], 3 with picornavirus, 2 with adenovirus, 1 with both influenza A virus and coronavirus [seasonal], 1 with parainfluenza virus, and 1 with RSV) and were excluded from the comparative analysis. Furthermore, cases of FLUV coinfection with other viruses (n = 84) and RSV with other viruses (n = 31) were also excluded. Characteristics of coinfections are displayed in Supplementary Tables 1 and 2.

**Figure 2.**
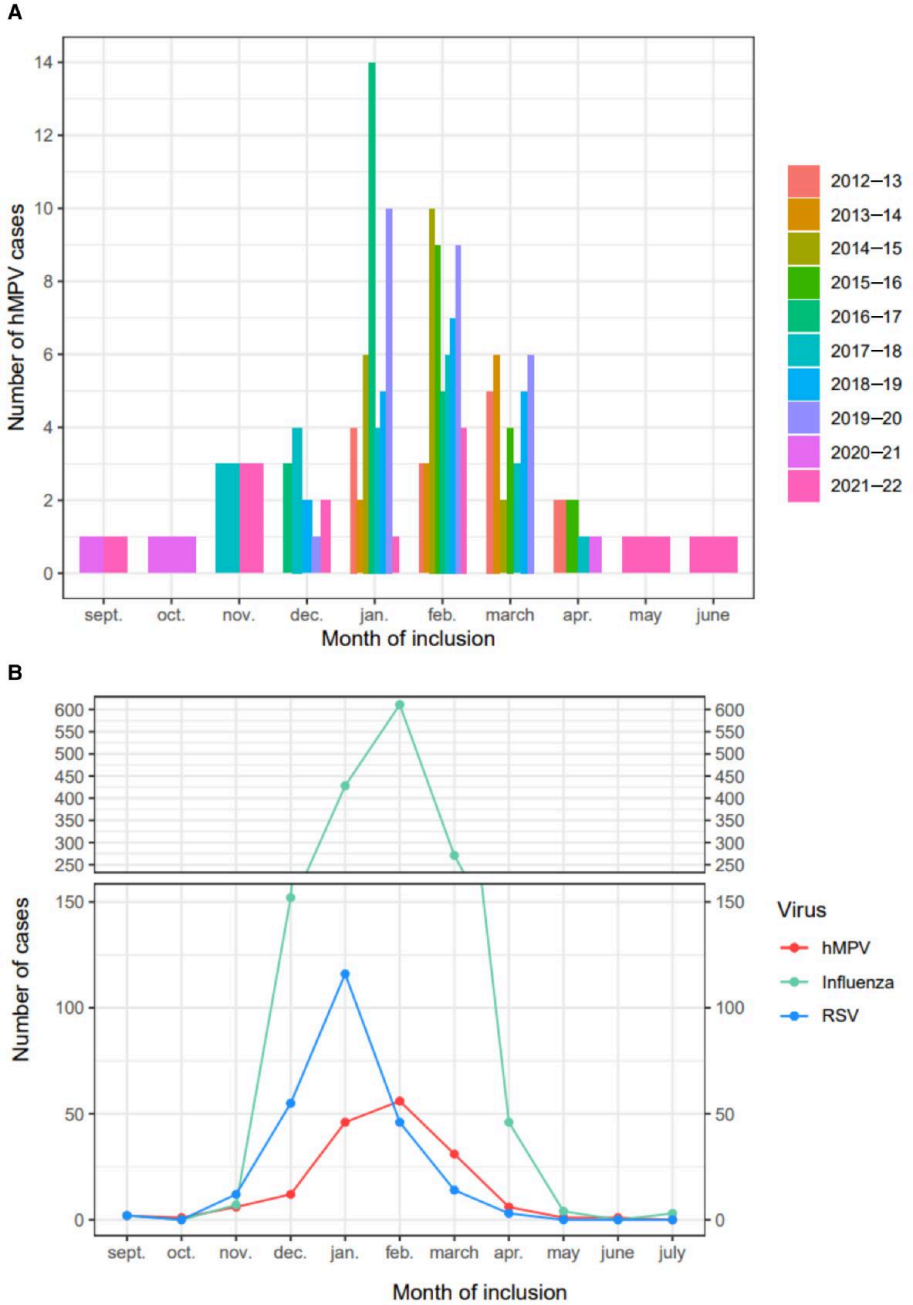
Human metapneumovirus (hMPV) monthly distributions according to the seasons of the FLUVAC study (*A*) and monthly distribution of hMPV, influenza virus, and respiratory syncytial virus (RSV) inclusions for all seasons (*B*). Inclusion periods: 21 November 2012 to 11 April 2013; 5 January 2014 to 2 April 2014; 6 January 2015 to 30 March 2015; 25 January 2016 to 27 April 2016; 15 December 2016 to 2 March 2017; 11 November 2017 to 12 April 2018; 26 October 2018 to 27 March 2019; 11 December 2019 to 16 March 2020; and 23 September 2020 to 28 September 2022.

Peak hMPV and FLUV detection occurred in February, while RSV occurred in January (Figure 2).

The overall rate of respiratory virus detection was slightly higher during the COVID-19 pandemic than before (58% vs 53%, *P* < .001). Still, the detection of hMPV decreased (like all other respiratory viruses) from 6% (range, 5%–8%) to 3% before and during the pandemic, respectively. hMPV prevalence was higher than RSV during the COVID-19 pandemic (Supplementary Table 3).

The overall seasonal influenza vaccine coverage was 50.5%. Patients who were vaccinated were older and more often had at least 1 chronic disease, primarily respiratory or cardiac disease, or were receiving immunosuppressive treatment (Supplementary Table 4).

### Description of hMPV^+^ Patients

Among patients with hMPV infection, 57% were female, with a median age of 78 years (IQR, 67–85 years). Most reported at least 1 chronic condition (81% [116/143]), mainly chronic cardiac (50% [72/143]) and respiratory (39% [56/143]) conditions; 15% were receiving immunosuppressive treatment (22/143), and 12% had cancer (17/143) (Table 1). In comparing the hMPV^+^ versus hMPV^–^ patients, only female sex and older age remained significant in multivariate analysis (Supplementary Table 5).

The main symptoms of hMPV^+^ patients at the time of inclusion were fever, cough, and shortness of breath. The median time from symptom onset to hospital admission was 2 days (IQR, 1–3 days).

The median length of stay was 7 days (IQR, 4–12 days), and nearly two-thirds (86/143 [60%]) of the patients had complications during the stay, mainly pneumonia (35% [45/128]) and exacerbation of chronic respiratory disease (30% [43/143]) or cardiac disease (22% [32/143]). Fifteen percent (22/143) were admitted to the intensive care unit (ICU), and 4% (6/143) died.

### Comparison of hMPV^+^ and FLUV^+^ Patients

Compared to FLUV^+^ patients, hMPV^+^ patients were older, more frequently vaccinated against influenza, had more chronic cardiac disease, and more likely to present with dyspnea but less likely to present with weakness, myalgia, headache, and sore throat. They were more likely to develop at least 1 complication (60% vs 50%, *P* = .02), especially acute heart failure, which was twice as common in hMPV^+^ during the hospital stay (22% vs 11%, *P* < .001), and resulted in a longer length of stay (median, 7 [IQR, 4–12] days vs 6 [IQR, 3–10] days; *P* = .01) (Table 1). Notably, these differences were more pronounced among patients who had been vaccinated against influenza, indicating a reduction in the severity of outcomes for influenza-vaccinated patients (Supplementary Table 6).

### Comparison of hMPV^+^ and RSV^+^ Patients

In comparison to RSV^+^ patients, hMPV^+^ patients were less likely to have chronic respiratory diseases (*P* = .02) and cancer (*P* = .04), less frequently presented with dyspnea and weakness at inclusion, and were less frequently admitted to ICU (15% vs 25%, *P* = .03). The mortality rate was similar (4% vs 5%) (Table 1).

### Comparison of hMPV^+^ and Patients Without Any Respiratory Virus Found

Compared to patients without any respiratory virus, hMPV^+^ patients were older. They were less likely to have weakness but more likely to show fever and cough at inclusion. They experienced more complications during their stay (60% vs 52%, *P* = .05), primarily respiratory failure and acute heart failure (Table 1).

The in-hospital all-cause death rate was similar among all populations (4% hMPV^+^, 4% FLUV^+^, 5% RSV^+^, and 5% in those without respiratory viruses).

No differences were found in the sensitivity analyses that included coinfections (data not shown).

## DISCUSSION

In our analysis of 6599 hospitalized adult patients with community-acquired ILI, half had at least 1 respiratory virus. Due to the nonpharmacological containment measures, the detection rate of all non-SARS-CoV-2 respiratory viruses, including hMPV, decreased sharply during the COVID-19 pandemic. Overall, hMPV, RSV, and FLUV were found in 5%, 7%, and 42% of those with at least 1 respiratory virus, respectively (6%, 10%, and 65% when excluding the COVID-19 period). These prevalences are consistent with several studies that found hMPV in 3%–6% of adults hospitalized for acute respiratory infection [9, 10]. Most hMPV cases were found in December and January, consistent with the inclusion period during influenza circulation in 8 of the 10 years of recruitment. Interestingly, some cases were found in autumn and spring in the last 2 years with year-round inclusion. The changes in seasonality after the lifting of social distancing restrictions might have favored this off-peak detection.

Most hMPV^+^ patients presented with respiratory symptoms and fever, whereas general symptoms (weakness, headaches, and myalgias) were less common (20% or less). This clinical presentation was similar to that of RSV^+^ patients but differed from that of FLUV^+^ patients, who had more general symptoms. These results align with the recent Hospitalized Acute Respiratory Tract Infection (HARTI) study, which suggested that symptom severity for RSV and hMPV may be greater than for FLUV at inclusion and during hospitalization, particularly for older adults and those with chronic conditions [10].

The hMPV^+^ hospitalized adults were old and had a high rate of chronic conditions, especially chronic cardiac and respiratory disease, which is consistent with the existing literature [11–14]. The rate of chronic cardiac condition was similar in RSV^+^ patients but higher than in FLUV^+^ patients.

We observed a significantly higher rate of influenza vaccination in hMPV^+^ and RSV^+^ patients compared to FLUV^+^ patients. This may be explained by their higher rate of chronic conditions and the efficacy of the influenza vaccine in preventing hospitalization for influenza [15].

Complications were most frequent in hMPV^+^ patients (60%), especially pneumonia (1 of 3 patients) and acute heart failure (1 of 5 patients). The rate of heart failure was higher in hMPV^+^ patients than in FLUV^+^ patients but similar to that of RSV^+^ patients. The association between RSV^+^ or FLUV^+^ infections and acute cardiac events is well established, with an increased risk for acute coronary syndrome or heart failure exacerbation after RSV or influenza infection [16, 17] and a higher risk of RSV-associated hospitalization in patients with chronic heart failure [18]. We found a strong relationship between hMPV and cardiac conditions or events that warrants further exploration.

ICU admission (15%) and death (4%) in hMPV^+^ patients were similar to those reported with hMPV in a prospective international study from Falsey et al in 2017–2019 (15% ICU and 2% death) [19].

Mortality rates were similar among virus-positive patients in our study, consistent with the study of Boon et al in the Netherlands, which reported no difference between viruses as well but a higher rate of mortality (around 10%) [20].

This study, which included 3455 additional patients over 4 years, of whom 62 were hMPV^+^, confirms our previous findings on the characteristics and outcomes of hMPV compared to FLUV and RSV patients [21].

Our study's strengths include its multicenter design, the systematic inclusion of all patients with ILI, and the year-round inclusion for the last 2 seasons.

Our study had limitations. First, we enrolled patients only during influenza virus circulation in the first 8 seasons, which may not fully cover hMPV circulation. Second, systematic testing for viruses other than SARS-CoV-2 was less frequent during the last 2 seasons, especially in the early stages of the pandemic, due to organizational issues (Supplementary Table 3). These 2 points might have led to an underestimation of hMPV incidence. Third, other pathogens, such as respiratory bacteria, may have been involved, and in coinfections, hMPV might have been a concomitant infectious agent with no role in the reported symptoms. The absence of data on bacteriological results prevented us from addressing this issue. Finally, the limited number of hMPV infections may have hindered our ability to perform a sufficiently powered comparison to reveal significant differences with the other viruses.

In conclusion, although hMPV infections were less prevalent than influenza and RSV in hospitalized adults, they significantly burden healthcare resources. These infections primarily affect older patients with chronic conditions, leading to pulmonary and cardiac complications. To protect these vulnerable populations, who are already more likely to be vaccinated against influenza and will soon be vaccinated against RSV, a global strategy for preventing respiratory viral infections through the development of hMPV vaccines and antivirals should be considered.

## Supplementary Material

jiaf082_Supplementary_Data

## Supplementary Data

Supplementary materials are available at *The Journal of Infectious Diseases* online (http://jid.oxfordjournals.org/). Supplementary materials consist of data provided by the author that are published to benefit the reader. The posted materials are not copyedited. The contents of all supplementary data are the sole responsibility of the authors. Questions or messages regarding errors should be addressed to the author.

## Notes

***Author contributions.*** P. L. and O. L. conceptualized the study. P. L., C. D., and L. M. developed the methodology. S. G. and C. D. analyzed the data. P. L. wrote the original manuscript. P. V., F. L., F. G., X. D., B. L., M. V., G. L., V. F., N. F., A.S. L., S. R., L. H. L., L. B. L. N., N. H.-F., and F. C. and L. M. critically reviewed the manuscript.

***Disclaimer.*** Vaccine producers had no role in the study design, data analysis, publication decision, or manuscript preparation.

***Financial support.*** Agence Nationale de Recherche contre le Sida et les/Maladies Infectieuses Emergentes funded S. G.'s salary. During the FLUVAC study years, the study sites received funding from the I-REIVAC network as part of the DRIVE and I-MOVE programs and from Janssen.

***Supplement sponsorship.*** This article appears as part of the supplement “Human Metapneumovirus Disease Burden in Older Adults and Implications for Adult Vaccination Strategies,” sponsored by AstraZeneca.
