# Supplementary material for: Characteristics of Human Metapneumovirus Infection Compared to Respiratory Syncytial Virus and Influenza Infections in Adults Hospitalized for Influenza-Like Illness in France, 2012–2022
Source: J Infect Dis. 2025 Jul 16;232(Suppl 1):S93–S100. doi: 10.1093/infdis/jiaf082 (PMC12265065; doi:10.1093/infdis/jiaf082)
Supplement: jiaf082_Supplementary_Data [file jiaf082_supplementary_data.docx]

**Supplementary Data**

**Supplementary table 1. Description of co-infections**

|  | **Co-infections hMPV,**  **N = 19** | **Co-infections FLUV,**  **N = 84** | **Co-infections RSV,**  **N = 31** |
| --- | --- | --- | --- |
| Adenovirus | 2 (11%) | 9 (11%) | 1 (3%) |
| Bocavirus | - | 14 (17%) | 1 (3%) |
| Influenza (untyped) | 1 (5%) | - | - |
| Influenza A | 3 (16%) | - | 13 (42%) |
| Influenza A, Seasonal Coronavirus | 1 (5%) | - | - |
| Influenza B | - | - | 3 (10%) |
| Parainfluenza | 1 (5%) | 3 (4%) | - |
| Picornavirus | 3 (16%) | 15 (18%) | 5 (16%) |
| Picornavirus, Seasonal Coronavirus | - | 3 (4%) | 1 (3%) |
| RSV | 1 (5%) | 16 (19%) | - |
| SARS-CoV-2 | 3 (16%) | 8 (10%) | - |
| Seasonal Coronavirus | 4 (21%) | 16 (19%) | 7 (23%) |

**Supplementary table 2. Characteristics of co-infection**

|  | **Co-infection hMPV**  **(n=19)** | **Co-infection FLUV**  **(n=84)** | **Co-infection RSV**  **(n=31)** |
| --- | --- | --- | --- |
| **Baseline characteristics** |  |  |  |
| **Gender** |  |  |  |
| Women, n (%) | 13/19 (68%) | 32/84 (38%) | 14/31 (45%) |
| **Age** |  |  |  |
| Median age, years (IQR) | 75 (64 - 83) | 66 (54 - 81) | 70 (59 - 77) |
| **Age group** |  |  |  |
| 18-49 years old, n (%) | 2/19 (11%) | 16/84 (19%) | 3/31 (10%) |
| 50-64 years old, n (%) | 3/19 (16%) | 25/84 (30%) | 10/31 (32%) |
| 65-74 years old, n (%) | 3/19 (16%) | 9/84 (11%) | 9/31 (29%) |
| > 74 years old, n (%) | 11/19 (58%) | 34/84 (40%) | 9/31 (29%) |
| **Median BMI**, kg/m2 (IQR) | 25 (21 – 30) | 23.7 (20.9 – 27.8) | 24 (21 – 30) |
| **Smoking status** |  |  |  |
| Smoker, n (%) | 3/17 (18%) | 19/78 (24%) | 6/31 (19%) |
| Ex-smoker, n (%) | 3/17 (18%) | 18/78 (23%) | 9/31 (29%) |
| Non-smoker, n (%) | 11/17 (65%) | 41/78 (53%) | 16/31 (52%) |
| **Chronic diseases** (at least one), n (%) | 15/19 (79%) | 60/84 (71%) | 28/31 (90%) |
| Chronic respiratory disease, n (%) | 9/19 (47%) | 30/84 (36%) | 16/31 (52%) |
| Chronic heart disease, n (%) | 10/19 (53%) | 33/84 (39%) | 18/31 (58%) |
| Diabetes, n (%) | 4/19 (21%) | 13/84 (15%) | 9/31 (29%) |
| Chronic renal failure, n (%) | 3/19 (16%) | 12/84 (14%) | 7/31 (23%) |
| Cancer, n (%) | 2/19 (11%) | 13/84 (15%) | 9/31 (29%) |
| Cirrhosis, n (%) | 0/19 (0%) | 0/84 (0%) | 3/31 (10%) |
| **Immunosuppressive treatment, n (%)** | 4/19 (21%) | 16/82 (20%) | 9/30 (30%) |
| **Influenza vaccination, n (%)** | 14/19 (74%) | 41/83 (49%) | 17/31 (55%) |
| **Hospitalization in the previous 12 months, n (%)** | 8/19 (42%) | 31/84 (37%) | 18/31 (58%) |
| **Presence of child/children <5 in the household, n (%)** | 1/19 (5%) | 7/84 (8%) | 1/31 (3%) |
| **Clinical presentation** |  |  |  |
| **Median time from symptom onset to hospitalization, days (IQR)** | 3 (1 – 4) | 2 (1 – 3) | 2 (1 – 5) |
| **Symptoms** |  |  |  |
| Fever or feverishness, n (%) | 15/19 (79%) | 74/84 (88%) | 28/31 (90%) |
| Cough, n (%) | 15/19 (79%) | 69/84 (82%) | 29/31 (94%) |
| Dyspnea, n (%) | 13/16 (81%) | 49/61 (80%) | 21/27 (78%) |
| Weakness/malaise, n (%) | 2/19 (11%) | 17/83 (20%) | 5/31 (16%) |
| Headache, n (%) | 5/19 (26%) | 24/83 (29%) | 7/31 (23%) |
| Myalgia, n (%) | 4/19 (21%) | 22/83 (27%) | 10/31 (32%) |
| Sore throat, n (%) | 5/19 (26%) | 21/83 (25%) | 7/31 (23%) |
| **Outcomes** |  |  |  |
| **At least one complication during the hospital stay, n (%)** | 10/19 (53%) | 41/84 (49%) | 14/31 (45%) |
| Pneumonia, n (%) | 3/16 (19%) | 17/79 (22%) | 6/28 (21%) |
| Respiratory failure, n (%) | 4/19 (21%) | 17/84 (20%) | 5/31 (16%) |
| Acute heart failure, n (%) | 4/19 (21%) | 11/84 (13%) | 3/31 (10%) |
| Acute respiratory distress syndrome, n (%) | 0/19 (0%) | 6/84 (7%) | 3/31 (10%) |
| **Median length of stay, days (IQR)** | 8 (3.5 – 12.5) | 6 (3 – 10) | 7 (5 – 12) |
| **ICU admission, n (%)** | 3/19 (16%) | 10/84 (12%) | 5/31 (16%) |
| **Mechanical ventilation, n (%)** | 3/14 (21%) | 7/49 (14%) | 3/23 (13%) |
| Invasive, n (%) | - | 0/2 (0%) | 0/1 (0%) |
| Non-invasive, n (%) | - | 2/2 (100%) | 1/1 (100%) |
| **Extracorporeal membrane oxygenation, n (%)** | 0/14 (0%) | 0/49 (0%) | 0/23 (0%) |
| **All-cause Death, n (%)** | 0/19 (0%) | 2/84 (2%) | 1/31 (3%) |

**Supplementary table 3. Rate of detection of respiratory viruses before and during the COVID-19 pandemic.**

| **Viruses** |  | **Before COVID-19**  **(13/14-19/20)**  **N= 3932** | **During COVID-19**  **(20/21-21/22)**  **N=2667** | **Total**  **N= 6599** |
| --- | --- | --- | --- | --- |
| At least one virus |  | 2077 (53%) | 1543 (58%) | 3620 (55%) |
| Influenza |  | 1358/2077 (65%) | 166/1542 (11%) | 1524/3619 (42%) |
| *Influenza A* |  | 1006/1337 (75%) | 140/160 (88%) | 1146/1497 (77%) |
| *Influenza B* |  | 331/1337 (25%) | 20/160 (12%) | 351/1497 (23%) |
| Picornavirus |  | 247/2070 (12%) | 57/1416 (4%) | 304/3486 (9%) |
| RSV |  | 216/2072 (10%) | 32/1416 (2%) | 248/3488 (7%) |
| Seasonal Coronavirus |  | 149/1339 (11%) | 40/1453 (3%) | 189/2792 (7%) |
| *SARS-CoV-2* |  | - | 1226/1453 (84%) | 1226/2792 (44%) |
| hMPV |  | 121/2047 (6%) | 41/1416 (3%) | 162/3463 (5%) |
| Adenovirus |  | 44/2048 (2%) | 5/1424 (0%) | 49/3472 (1%) |
| Parainfluenza |  | 30/1852 (2%) | 4/1416 (0%) | 34/3268 (1%) |
| Bocavirus |  | 27/2072 (1%) | 3/1424 (0%) | 30/3268 (1%) |

**Supplementary table 4. Characteristics of participants who received the seasonal influenza vaccine compared to those who did not, 2012-2022.**

|  | **Influenza Unvaccinated**  **(n=3418)** | **Influenza Vaccinated**  **(n=3094)** | **p-value** |
| --- | --- | --- | --- |
| **Baseline characteristics** |  |  |  |
| **Gender** |  |  |  |
| Women, n (%) | 1598/3418 (47%) | 1378/3094 (45%) | 0,08 |
| **Age** |  |  |  |
| Median age, years (IQR) | 63 (49 - 76) | 78 (68 - 85) | <0,001 |
| **Age group** |  |  | <0,001 |
| 18-49 years old, n (%) | 855/3418 (25%) | 207/3094 (7%) |  |
| 50-64 years old, n (%) | 920/3418 (27%) | 393/3094 (13%) |  |
| 65-74 years old, n (%) | 692/3418 (20%) | 671/3094 (22%) |  |
| > 74 years old, n (%) | 951/3418 (28%) | 1823/3094 (59%) |  |
| **Median BMI**, kg/m2 (IQR) | 25 (22 - 30) | 25 (22 - 29) | 0,17 |
| **Smoking status** |  |  | <0,001 |
| Smoker, n (%) | 767/3366 (23%) | 370/3017 (12%) |  |
| Ex-smoker, n (%) | 840/3366 (25%) | 1077/3017 (36%) |  |
| Non-smoker, n (%) | 1759/3366 (52%) | 1570/3017 (52%) |  |
| **Chronic diseases** (at least one), n (%) | 2279/3415 (67%) | 2679/3091 (87%) | <0,001 |
| Chronic respiratory disease, n (%) | 1116/3417 (33%) | 1447/3094 (47%) | <0,001 |
| Chronic heart disease, n (%) | 973/3416 (28%) | 1521/3094 (49%) | <0,001 |
| Diabetes, n (%) | 683/3417 (20%) | 844/3094 (27%) | <0,001 |
| Chronic renal failure, n (%) | 438/3417 (13%) | 553/3094 (18%) | <0,001 |
| Cancer, n (%) | 482/3417 (14%) | 584/3092 (19%) | <0,001 |
| Cirrhosis, n (%) | 135/3416 (4%) | 148/3093 (5%) | 0,10 |
| **Immunosuppressive treatment, n (%)** | 490/3395 (14%) | 521/3073 (17%) | 0,005 |
| **Hospitalization in the previous 12 months, n (%)** | 1215/3398 (36%) | 1489/3073 (48%) | <0,001 |
| **Presence of child/children <5 in the household, n (%)** | 287/3402 (8%) | 125/3,080 (4%) | <0,001 |

**Supplementary table 5. Factors associated with hMPV detection (hMPV+ vs. hMPV-) in hospitalized patients for influenza-like illness and tested for hMPV, 2012-2022.**

|  | **Univariable analysis,**  **N = 4076** | | **Multivariable analysis,**  **N = 3936** | |
| --- | --- | --- | --- | --- |
|  | **OR** | **p-value** | **aOR** | **p-value** |
| **Gender** |  | 0.01 |  | 0.05 |
| Women, n = 1852 | Ref |  | Ref |  |
| Men, n = 2224 | 0.65 (0.46; 0.92) |  | 0.69 (0.47; 0.99) |  |
| **Age group** |  | <0.01 |  | <0.01 |
| 18-49 years, n = 686 | Ref |  | Ref |  |
| 50-64 years, n = 809 | 2.32 (1.06; 5.09) |  | 2.58 (1.17; 5.71) |  |
| 65-74 years, n = 861 | 2.60 (1.21; 5.59) |  | 2.97 (1.34; 6.56) |  |
| >74 years, n = 1720 | 3.70 (1.83; 7.46) |  | 3.73 (1.77; 7.87) |  |
| **BMI, n = 4010** | 1.00 (0.99; 1.01) | >0.99 |  |  |
| **Smoking status** |  | 0.08 |  | 0.55 |
| Non-smoker, n = 2040 | Ref |  | Ref |  |
| Ex-smoker, n = 1127 | 0.82 (0.54; 1.24) |  | 0.91 (0.58; 1.41) |  |
| Smoker, n = 813 | 0.57 (0.34; 0.94) |  | 0.74 (0.42; 1.28) |  |
| **Chronic diseases** (at least one, n = 3196) |  | 0.18 |  | 0.10 |
| None, n = 874 | Ref |  | Ref |  |
| Cardio-respiratory and other diseases^1^ (at least one, n = 1291) | 0.88 (0.53; 1.46) |  | 0.64 (0.37; 1.11) |  |
| Other diseases^1^ (at least one, n = 505) | 0.86 (0.44; 1.69) |  | 0.70 (0.34; 1.43) |  |
| Cardio-respiratory diseases (at least one, n = 1400) | 1.00 (0.99; 1.01) |  | 1.07 (0.66; 1.75) |  |
| **Immunosuppressive treatment**, n = 703 (vs. no, n = 3349) | 0.85 (0.53; 1.35) | 0.49 | 0.95 (0.58; 1.57) | 0.85 |
| **Influenza vaccination**, n = 1944 (vs. no, n = 2105) | 1.29 (0.91; 1.82) | 0.15 | 1.07 (0.74; 1.56) | 0.71 |
| **Hospitalization in the previous 12 months**, n = 1810 (vs. no, n = 2252) | 0.83 (0.59; 1.18) | 0.30 | 0.81 (0.57; 1.17) | 0.27 |

***^1^****Other diseases: diabetes, renal disease, cancer or cirrhosis*

**Supplementary table 6. Characteristics and outcome of hospitalized patients infected with human Metapneumovirus and influenza according to the influenza vaccine status, 2012-2022, after exclusion of co-infections**

|  | **Influenza vaccinated** | | | **Influenza unvaccinated** | | |
| --- | --- | --- | --- | --- | --- | --- |
|  | **hMPV+**  **(n=81)** | **FLUV+**  **(n=615)** | **p-value** | **hMPV+**  **(n=61)** | **FLUV+**  **(n=811)** | **p-value** |
| **Baseline characteristics** |  |  |  |  |  |  |
| **Gender** |  |  |  |  |  |  |
| Women, n (%) | 48/81 (59%) | 287/615 (47%) | 0.03 | 33/61 (54%) | 419/811 (52%) | 0.79 |
| **Age** |  |  |  |  |  |  |
| Median age, years (IQR) | 80 (72 - 88) | 78 (68 - 85) | 0.03 | 72 (60 - 80) | 63 (48 - 75) | 0.002 |
| **Age group** |  |  | 0.28 |  |  | 0.008 |
| 18-49 years old, n (%) | 2/81 (2%) | 45/615 (7%) |  | 7/61 (11%) | 223/811 (27%) |  |
| 50-64 years old, n (%) | 7/81 (9%) | 76/615 (12%) |  | 16/61 (26%) | 210/811 (26%) |  |
| 65-74 years old, n (%) | 17/81 (21%) | 121/615 (20%) |  | 11/61 (18%) | 159/811 (20%) |  |
| > 74 years old, n (%) | 55/81 (68%) | 373/615 (61%) |  | 27/61 (44%) | 219/811 (27%) |  |
| **Median BMI**, kg/m2 (IQR) | 25.6 (21.6 - 29.3) | 24.7 (21.9 - 28.4) | 0.41 | 24 (21 - 30) | 25 (22 - 29) | 0.90 |
| **Smoking status** |  |  | 0.03 |  |  | 0.02 |
| Smoker, n (%) | 7/79 (9%) | 77/593 (13%) |  | 13/60 (22%) | 208/795 (26%) |  |
| Ex-smoker, n (%) | 19/79 (24%) | 216/593 (36%) |  | 22/60 (37%) | 161/795 (20%) |  |
| Non-smoker, n (%) | 53/79 (67%) | 300/593 (51%) |  | 25/60 (42%) | 426/795 (54%) |  |
| **Chronic diseases** (at least one), n (%) | 75/81 (93%) | 527/614 (86%) | 0.12 | 40/61 (66%) | 558/810 (69%) | 0.57 |
| Chronic respiratory disease, n (%) | 36/81 (44%) | 272/615 (44%) | 1.0 | 20/61 (33%) | 283/811 (35%) | 0.78 |
| Chronic heart disease, n (%) | 46/81 (57%) | 312/615 (51%) | 0.35 | 26/61 (43%) | 254/811 (31%) | 0.09 |
| Diabetes, n (%) | 23/81 (28%) | 167/615 (27%) | 0.79 | 8/61 (13%) | 160/811 (20%) | 0.24 |
| Chronic renal failure, n (%) | 11/81 (14%) | 98/615 (16%) | 0.75 | 8/61 (13%) | 84/811 (10%) | 0.52 |
| Cancer, n (%) | 10/81 (12%) | 97/615 (16%) | 0.51 | 6/61 (10%) | 112/811 (14%) | 0.56 |
| Cirrhosis, n (%) | 5/81 (6%) | 18/614 (3%) | 0.17 | 1/61 (2%) | 34/810 (4%) | 0.51 |
| **Immunosuppressive treatment, n (%)** | 9/81 (11%) | 96/613 (16%) | 0.33 | 13/61 (21%) | 132/809 (16%) | 0.29 |
| **Hospitalization in the previous 12 months, n (%)** | 35/81 (43%) | 278/613 (45%) | 0.81 | 23/61 (38%) | 307/811 (38%) | 1.0 |
| **Presence of child/children <5 in the household, n (%)** | 1/81 (1%) | 36/615 (6%) | 0.11 | 6/61 (10%) | 79/811 (10%) | 1.0 |
| **Outcomes** |  |  |  |  |  |  |
| **At least one complication during the hospital stay, n (%)** | 51/81 (63%) | 312/615 (51%) | 0.04 | 34/61 (56%) | 397/811 (49%) | 0.35 |
| Pneumonia, n (%) | 27/76 (36%) | 156/587 (27%) | 0.10 | 17/51 (33%) | 221/769 (29%) | 0.52 |
| Respiratory failure, n (%) | 23/81 (28%) | 123/615 (20%) | 0.08 | 19/61 (31%) | 189/811 (23%) | 0.16 |
| Acute heart failure, n (%) | 23/81 (28%) | 87/615 (14%) | 0.002 | 8/61 (13%) | 70/810 (9%) | 0.24 |
| Acute respiratory distress syndrome, n (%) | 5/81 (6%) | 44/615 (7%) | 1.0 | 4/61 (7%) | 76/811 (9%) | 0.65 |
| **Median length of stay, days (IQR)** | 9 (5 - 13) | 6 (3 - 11) | 0.008 | 6 (4 - 9) | 5 (3 -z 10) | 0.69 |
| **ICU admission, n (%)** | 12/81 (15%) | 87/615 (14%) | 0.87 | 10/61 (16%) | 153/811 (19%) | 0.74 |
| **Mechanical ventilation, n (%)** | 6/51 (12%) | 45/365 (12%) | 1.0 | 5/37 (14%) | 57/391 (15%) | 1.0 |
| Invasive, n (%) | 0/1 (0%) | 2/14 (14%) | 1.0 | 1/1 (100%) | 2/8 (25%) | 0.33 |
| Non-invasive, n (%) | 1/1 (100%) | 12/14 (86%) | 1.0 | 0/1 (0%) | 8/8 (100%) | 0.11 |
| **Extracorporeal membrane oxygenation, n (%)** | 0/51 (0%) | 0/365 (0%) | 1.0 | 0/37 (0%) | 3/391 (1%) | 1.0 |
| **All-cause Death, n (%)** | 5/81 (6%) | 18/615 (3%) | 0.17 | 1/61 (2%) | 30/809 (4%) | 0.72 |
